# Clients’ satisfaction with quality of childbirth services: A comparative study between public and private facilities in Limuru Sub-County, Kiambu, Kenya

**DOI:** 10.1371/journal.pone.0193593

**Published:** 2018-03-14

**Authors:** Clarice Okumu, Boniface Oyugi

**Affiliations:** 1 Reproductive and Maternal Services Unit–Division of Family Health, Ministry of Health, Nairobi, Kenya; 2 University of Nairobi, School of Public Health, Health Systems Management, Nairobi, Kenya; 3 Centre for Health Services Studies (CHSS), University of Kent, Canterbury, England; TNO, NETHERLANDS

## Abstract

**Background:**

This study intended to compare the clients’ satisfaction with the quality of childbirth services in a private and public facility amongst mothers who have delivered within the last twenty four to seventy hours.

**Methods:**

This was a cross-sectional comparative research design with both quantitative and qualitative data collection and analysis methods. Data were collected through a focused group discussion guide and structured questionnaire collecting information on clients’ satisfaction with quality of childbirth services. The study was conducted amongst women of reproductive age (WRA) between 15–49 years in Tigoni District hospital (public hospital) and Limuru Nursing home (private hospital). For quantitative data we conducted descriptive analysis and Mann-Whitney test using SPSS version 20.0 while qualitative data was manually analyzed manually using thematic analysis.

**Results:**

A higher proportion of clients from private facility 98.1% were attended within 0–30 minutes of arrival to the facility as compared to 87% from public facility. The overall mean score showed that the respondents in public facility gave to satisfaction with the services was 4.46 out of a maximum of 5.00 score while private facility gave 4.60. The level of satisfaction amongst respondents in the public facility on pain relief after delivery was statistically significantly higher than the respondents in private facilities (U = 8132.50, p<0.001) while the level of satisfaction amongst respondents in the public facility on functional equipment was statistically significantly higher than the respondents in private facilities (U = 9206.50, p = 0.001). Moreover, level of satisfaction with the way staff responded to questions and concerns during labour and delivery was statistically significantly higher than the respondents in private facilities (U = 9964.50, p = 0.022).

**Conclusion:**

In overall, majority of clients from both public and private facilities expressed satisfaction with quality of services from admission till discharge in both public and private facilities and were willing to recommend other to come and deliver in the respective facilities.

## Background

Satisfaction with healthcare services is defined as the extent to which the patients seeking treatment experience positive perception of the care provided by the nursing or medical staff. [1–3]. Patients’ satisfaction reveals the magnitude with which the healthcare needs are met and provides an essential gauge of high-quality healthcare which is used for the assessing and planning health interventions [4–7]. Ideally, patients who are satisfied with the care provided by the healthcare staff, are more likely to utilize health services in future and comply with the prescribed medical treatment to completion [3,8]. For patients to be more satisfied with treatment, there is need to provide high quality healthcare which is viewed as safe, timely, effective, efficient, equitable, and patient-centered [9]. Providing high quality of care in maternity services involves giving mothers the best possible medical care and outcome during antenatal, delivery, and postnatal period which can be measured against standard guidelines [10].

In accessing obstetric care, most clients are influenced by factors, such as a courteous provider attitude and competency, and availability of drugs and medical equipment, whereas cultural inappropriateness of care, disrespectful and inhumane services, and lack of emotional support can deter them from accessing obstetric care [8]. Provision of support, for instance, comfort and reassurance is beneficial and influences the mother’s assessment of quality [5,8]. However, perception of low quality has been reported as a major factor in non-utilization or bypassing of health services by patients [11]. In recent years, client satisfaction with clinical (process) services has gained recognition as an outcome of quality care [3]. Therefore, it is imperative to do a comprehensive review of the quality of healthcare during labour and delivery since most hospitals remain quiet on mechanisms of receiving feedback based on the perceptions of the patients [12].

There is mixed evidence in studies that have looked at the comparative analysis of the quality of service at private and public facilities. For instance, evidence shows that quality of care is low in both public and private facilities in developing countries although the private sector performs better than the public in terms of drug availability and responsiveness to clients’ needs [13,14]. Additionally, the private hospitals are considered better in regards to physical infrastructure and availability of services and are more efficient than the public health system; however, the difference between the two sectors is unnoticed in terms of technical quality of care provided [7,15]. According to Tuan et al, majority of the mothers choose to deliver in the private facilities than in nearby public facilities despite the fact that some public health facilities within the region are better equipped than the surrounding private facilities [7].

Other studies conducted have compared public and private hospitals that are in different level of organisation [1,2,14–16]. The levels of the hospital are defined by the facility perspective or the management type. In terms of the facility perspectives, the definitions are as follows: a) lowest level which has clinics, nursing homes, and dispensary; b) second level health centres; c) third level has county and sub-county hospitals; and d) the fourth level has the referral hospitals. On the other hand, the management levels are categorized as the government (GoK)/Public hospitals, Mission/Faith based organisation (FBO), Non-Governmental organisation (NGO), and private facilities. The other studies have also compared client’s satisfaction with other maternal services like antenatal care, family planning, and curative services between public and private facilities within different levels of organisation [14]. However, there is paucity of studies that compares the quality of child birth services (antenatal, perinatal, and post-natal) within level three healthcare facilities.

Therefore, in this paper we 1) compare the difference in the quality of child birth services at public and private facilities that are both level 3 facilities, 2) assess how the quality differentials impact upon client’s satisfaction with childbirth, 3) highlight women’s perception of care during labour delivery and the aspects of care which women consider important during childbirth, and 4) suggest quality improvements that can enhance child birth outcomes. This paper compares the clients’ satisfaction with the quality of childbirth services (antenatal, perinatal, and post-natal) amongst mothers who have delivered within the last twenty four to seventy two hours and ready for discharge in level 3 private and public facilities in Limuru, Kiambu County Kenya.

### Theoretical framework

The paper was based on Donabedian theory for examining health services and evaluating quality of care which allows insight into patient satisfaction at the various level of treatment [17]. According to the model, quality of care is drawn from three categories: structure (e.g., facilities, equipment, personnel, operational and financial processes supporting medical care, etc.), process (rely on the structures to provide resources and mechanisms for participants to carry out patient care activities), and outcomes (improve patient health in terms of promoting recovery, functional restoration, survival and even patient satisfaction) [9,17,18]. The framework is imperative in evaluating the following; a) differences and the similarities in the quality of care between public and private facilities b) client’s perception of quality in public and private facilities; and c) the relationship between client’s perception of quality of care and satisfaction with services.

In this paper, the variables from the framework which were used to measure how process services influence quality of childbirth services included–clients level of satisfaction with (outcome-dependent variable), turnaround time/waiting time (process-independent variable), treatment during labour and delivery (process-independent variable), privacy and confidentiality accorded during labour and delivery (process-independent variable) and information offered after delivery and before discharge (process-independent variable) as shown in Table 1.

**Table 1:** Donabedian model of measuring health care system performance.

Independent Variable	Service	Process	Dependent variable (Outcome)
**Treatment process**	Diagnosis of Pain and any other health condition before during labour and after delivery.	Prompt diagnosis and provision of adequate drug to control pain during labour and delivery leads client’s satisfaction.	Client satisfaction with quality of care
**Stages of treatment**	Pain relief during labour, Pain relief after delivery, and Emotional Support.	Use of efficacious drugs during labour and delivery will make clients comfortable, satisfied and may develop interest in use of the same facility or recommend it to another patient. Offering emotional support to a mother in labour by midwife or birth attendant yield better outcome of labour	
**Appropriateness**	Health Provider Technical competence in care of clients/patients	Use of the equipment in the health facility for detecting women’s and baby’s condition during labour and delivery and after delivery will lead to a successful outcome for mother and baby	
	Privacy and confidentiality	Maintaining privacy during procedures like examination during labour and delivery and asking them questions or responding to their needs in confidence boost clients self-esteem and will influence their level of satisfaction	
	Emotional support during labour and delivery	The extent to which clients /patients emotional needs are met during labour and delivery leads to confidence, self-esteem of the client satisfaction.	
	Care by provision of information after delivery and on discharge.	The prompt care after delivery which includes provision, which danger signs to observe in self and in baby, information on self-care and care of the baby has a role in influencing clients’ satisfaction with the services.	
**Service process**	Timeliness (waiting time.)	Attending the Clients/Patient promptly reduces unnecessary delays which may result into adverse outcomes and this positively influences overall satisfaction with care.	

## Methodology

### Study design and setting

This was a cross-sectional comparative research design where quantitative and qualitative data collection method was adopted. The qualitative methods were collected using a structured questionaire (discussed later in this paper) which comprised socio-demographic data and satisfaction with quality of child birth services questions and intended to provide a comprehensive picture of how the existing services met the needs of the population. On the other hand, the qualitative data collection method utilised was focused group discussion (also discused later in this paper) which was intended to provide to in-depth clarification of reasons of satisfaction and dissatisfaction by patients.The study was conducted between 16/04/2015 to 30/06/2015 in two study sites namely Tigoni Sub-County hospital (public hospital) and Limuru Nursing home (private hospital) which are in Limuru Sub-County in Kiambu, Kenya. Limuru Sub-County, which is one of the twelve sub-counties in Kiambu county, was selected randomly based on ease of access and convinience for the study team. The two hospitals were chosen because they are the only facilities offering Comprehensive Emergency Obstetric and Neonatal care facilities (CEmONC) in Limuru Sub-County. Tigoni is a public facility which offers curative, preventive and promotive health services and acts as a referral facility for Limuru sub-county and clients are referred from the lower level facilities within the catchment area for special care. Due to its proximity to Nakuru–Nairobi Highway it also serves emergency patients and any other patient who is not a resident of the area but has presented him/herself to the hospital without referral. The facility is also used as a training facility for student nurses, clinical officers and doctors who are on internship or attachment and has a catchment area population of 56,691 with 2,239 deliveries conducted between 2013–2014 [19]. On the other hand, Limuru Nursing home which is a privately owned facility within Limuru town central business division,offers curative, preventive and promotive services and serves all clients/patients who present there by choice. The catchment area population of Limuru Nursing Home is 33,810 with 1,222 deliveries conducted between 2013–2014 [19].

### Study participants and sampling

The study participants were women of reproductive age (WRA) between 15–49 years who delivered in Tigoni District Hospital and Limuru Nursing Home. For the exit interviews, the study included all mothers who delivered normally in both facilities. Only the mothers who had delivered within the last twenty four to seventy two hours and had recovered and ready for discharge were interviewed. Mothers who were below 18 years gave their accent but also had consent obtained from the guardian or husband for those who were already married. Women who had experienced stillbirths or had early neonatal deaths were excluded. Additionally, in the study we conducted one focus group discussion (FGD) comprising of 8 and 7 clients for Tigoni District Hospital and Limuru Nursing Home respectively. The mothers who were included in the FGDs were different from the mothers who were included in the exit interviews. The method to determine sample size was derived using Fisher’s et al. formula n = Z^2^ pq/d^2^ which is usually used for cross sectional studies [3] where n = the desired sample size, Z = the normal standard deviation, p = proportion in the target population estimated to have characteristics being measured, q = Proportion of population being measured and d = Level of statistical significance. A total of 307 mothers were targeted for inclusion in the study. The sample size was based on the prevalence of the health facility in Kiambu county using estimates from the Kiambu County Integrated Development Plan [20]. The sample size was allocated proportionally to each of the hospitals by reviewing the number of deliveries attended in financial year 2013–2014 (108 from Limuru Nursing Home and, 199 from Tigoni Sub County Hospital). The response rate was 97.7%. Simple random sampling technique was used to select clients for the interview each day of the study until the required sample size was fully achieved. The researchers used the Stat Trek's Random Number Generator to select the mothers. The Stat Trek's Random Number Generator used a statistical algorithm to produce random numbers and gave instructions on how to use it (*http://stattrek.com/statistics/random-number-generator.aspx*). The method allowed each mother to be interviewed only once after which the researcher hit the calculate button and the Random Number Generator produced a Random Number Table consisting of 15 random numbers between 1 and 30. The researcher then interviewed the mothers represented by these numbers which was done on daily basis depending on the number of mothers who had delivered every day in each of the two facilities until the right sample size was obtained.

### Data collection and analysis

Data collection was done using a structured questionnaire which was adapted from 4 previously used questionnaires [5,8,21,22]. The questions were selected in order of relevance and were used to measure clients’ level of satisfaction with waiting time, privacy and confidentiality, treatment and support during labour and delivery and information provided after delivery and before discharge. The questionnaire comprised socio-demographic data and satisfaction with quality of child birth services questions. The responses were presented using a 5 point Likert’s scale (1-Completely Dissatisfied/Disagree, 2-Dissatisfied/Disagree, 3-Not sure, 4-Satisfied/Agree, and 5-Completely Satisfied/Agree). One focus group discussions (FGD) was held in each facility and its aim was to to have in-depth clarification of reasons of satisfaction and dissatisfaction by clients so as to reinforce the quantitative data. The target patients for the FGD were mothers who had delivered but had not participated in the individual interviews. Each FGD lasted between one to two hours and field notes were taken. Besides, participation was through informed consent and was voluntary. A pilot study to pretest the data collection instrument was carried out on 10% of proposed research study population clients in Kiambu County Hospital and St.Teresa Nursing home (not included in the study) in order to identify any difficulties in understanding or completing the questionnaire and inorder to determine the point of saturation for the FGDs.

Data was cleaned, entered and analyzed using SPSS version 20.0 statistical package. The descriptive data was analyzed using frequency distribution and percentages. Chi-square was used to test for association while Mann-Whitney U test was used to test for the difference between two independent groups with the likert scores. P-value of 0.05 was taken for statistical significance. On the other hand, each FGD was conducted by two trained research assistants (one acted as the facilitatior and the other acted as the notes taker). Informed consent was obtained for all the participants. The discussions were recorded in the local language and then transcribed verbatim in the word format which was then tanslated to English. We did not conduct back translation of the transcripts into local language because of financial constrains. The transctibed work was then analysed manually using Excel 2010 by the two researchers.The data was coded and and the themes were then cartegorised within hierachical framework of main themes. The thematic framework was systematically applied to all transcripts. The associations and patterns of the themes were identified, and compared and contrasted amongst different respondents.

### Ethics

Ethical approval for the study was obtained from Great Lakes University of Kisumu Ethical Committee (GREC/192/02/2015). Permission was also obtained from the County and sub county health Executive Team (KBU/COUNTY/RESEARCH AUTHO/VOL 1/18). Written consent was obtained from the the respondents before they could participate in the study and confidentiality was ensured by protecting the identity of the participants at the point of data collection. Additionally, personal data was only accessible to trained data collectors, who had received training on ethical conduct prior to data collection, and the researcher. The respondents who were less than 18 years had the consent form signed by their guardians besides having a signed ascent form to participate in the study.

### Results

#### Socio-demographic characteristics

Majority of the respondents were aged between 15 and 24 years (45.1% in public and 48.6% in private facility), a sub-group that comprises youth and adolescents. Most of clients from both public (84.6%) and private (76.3%) facilities were in monogamous marriage. The largest part of respondents from both public and private facilities were Christian Protestants (public 71.3% and 76.2% in private) and a higher proportion of clients from both public (45.7%) and private facilities (41.5%) had attained secondary education. In terms of parity, majority of clients slightly over half (56.4%) from public and (50.5%) from private facilities had between 2 to 5 children. A higher proportion of clients from both public (44.6%) and private facilities (48.6%) were unemployed. With respect to respondents’ income, equal proportions of clients from public (46.7%) and private facilities (46.7%) had no source of income. There was no statistically significant difference between private and public facilities in the sociodemographic characteristics of respondents (Table 2).

**Table 2:** Association of socio-demographic characteristics of the respondents with choice of hospital.

		Public(n = 195)	Private (n = 105)	Chi-Square (χ2)	p-value
Age in completed years	15–24	88(45.1%)	51(48.6%)	2.323	0.803
	25–34	65(33.3%)	29(27.6%)		
	35–44	42(21.5%)	25(23.8%)		
	45+	0 (0%)	0 (0%)		
Marital Status	Single	27(13.8%)	25(23.8%)	6.117	0.106
	Married Monogamous	165(84.6%)	80 (76.2%)		
	Married Polygamous	2(1.0%)	0(0%)		
	Widowed	1(0.5%)	0(0%)		
Religion	Christian Protestant	139(71.3%)	80(76.2%)	4.547	0.337
	Christian Catholic	46(23.6%)	20(19.0%)		
	Muslim	1(0.5%)	1(0.5%)		
	No Religion	5(2.6%)	0(0%)		
	Other	4(2.1%)	4(3.8%)		
Education	No Education	3(1.5)	1(1.0%)	4.494	0.343
	Primary	73(37.4%)	43(41%)		
	Secondary	81(41.5%)	48(45.7%)		
	College	35(17.9%)	10(9.5%)		
	University	3(1.5)	3(2.9)		
Parity	Primigravida	77(39.5)	49(46.7)	1.569	0.456
	Para 2–5	108(56.8)	62(52.1)		
	Parity of above 5+	8(4.1)	3(2.9)		
Occupation	Student	7(3.6%)	7(6.7%)	2.327	0.507
	Unemployed	87(44.6%)	51(48.6%)		
	Self-Employed	68(34.9)	31(29.5%)		
	Salaried/Formal Employment	33(16.9)	16(15.2%)		
Income (In Kenya Shillings)	None	91(46.7%)	49(46.7%)	3.094	0.377
	1–5,000	50(25.6%)	35(33.3%)		
	5,001–10,000	37(19.0%)	15(14.3%)		
	Above 10,000	17(8.7%)	6(5.7%)		

### Time taken for clients to be attended

The results showed that there was a statistically significant difference in the time taken by a client to be attended at a public and private facility as shown in Table 3. A higher proportion of clients from private facility (98.1%) were attended within 0–30 minutes of arrival to the facility as compared to (87%) from public facility. However, the results from the FGD that asked the respondents to comment on the duration of time spent waiting to be attended showed that respondents in both hospitals indicated that they were all satisfied with the time taken as none stayed long in the waiting area as reported below.

**Table 3:** Difference in time taken for clients to be attended between public and private facility.

Variable	Facility	0–30 mins	30 min-1 hr.	1–2hrs	>2 hrs.	χ^2^	p-value
Time taken to be attended	Public n = 195	170(87%)	17(8.9%)	5(2.6%)	3(1.5%)	10.204	0.017**
	Private n = 105	103(98.1%)	2(1.9%)	0(0%)	0(0%)		

** denotes statistical significance between public and private facility at 95% CI. P-value computed using Chi-Square at P value <0.05.

“When I arrived, I took a short time, they acted very fast, I was taken to labour ward, I was examined and given details on my status and I felt that I was well treated” (Respondent public facility)

“When I came to the reception, I was immediately attended to and referred upstairs (to theatre), I was very happy and feel satisfied because I was not kept waiting and this is very encouraging”(Respondent Private facility)

### Satisfaction level of clients with the services

On the satisfaction level of the clients with the services, all the clients in the public (195) and private (105) facility gave their response to all the 23 parameters that they were asked to rate. The overall mean score the respondents in public facility gave to satisfaction with the services was 4.46 out of a maximum of 5.00 score (Table 4) while in private facility was 4.60 (Table 5).

**Table 4:** The proportions and mean satisfaction scores of respondents with the services in public facility.

Public			
Variable	Completely satisfied n (%)	Satisfied n (%)	Mean (SD)
Satisfaction with the waiting time	120 (63.2)	59 (31.1)	**4.51 (0.80)**
Regular observations during waiting time	127 (66.8)	52 (27.4)	**4.54 (0.82)**
Confidentiality of information	154 (81.1)	34 (17.9)	**4.79 (0.48)**
Privacy during vaginal examination	158 (83.2)	26 (13.7)	**4.77 (0.59)**
Privacy during delivery	140 (73.7)	41 (21.6)	**4.66 (0.68)**
Draping during delivery	137 (72.1)	45 (23.7)	**4.64 (0.70)**
Pain management during labour	58 (30.5)	84 (44.2)	**3.81(1.13)**
Pain management after delivery	103 (54.2)	65 (34.2)	**4.31 (0.96)**
Qualified health-workers	89 (46.8)	93 (48.9)	**4.38 (0.74)**
Functional equipment	121 (63.7)	67 (35.3)	**4.62 (0.56)**
Satisfaction with response from staff	153 (80.5)	30 (15.8)	**4.75 (0.61)**
Guidance to labour companion	3 (1.6)	5 (2.6)	**1.97 (0.30)**
Reception of labour companion	1 (0.5)	1 (0.5)	**2.50 (1.30)**
Support and encouragement during labour	102 (53.7)	53 (27.9)	**4.18 (1.11)**
Guidance during labour	138 (72.6)	37 (19.5)	**4.57 (0.85)**
Satisfaction with delivery	135 (71.1)	42 (22.1)	**4.57 (0.82)**
Encouragement to breastfeed	128 (67.4)	36 (18.9)	**4.41 (1.03)**
Provision of information on baby’s status	117 (61.6)	67 (35.3)	**4.55 (0.69)**
Information on danger signs after delivery	108 (56.8)	60 (31.6)	**4.34 (0.97)**
Information on danger signs on the baby	109 (57.4)	32 (16.8)	**4.06 (1.28)**
Information on self-care	106 (55.8)	65 (34.2)	**4.36 (0.90)**
Information on care of the baby	106 (55.8)	66 (34.7)	**4.37 (0.89)**
**Overall satisfaction**	**101 (53.2)**	**82 (43.2)**	**4.46 (0.70)**

**Note:** The table only reports two levels from the Likert scale (“Completely satisfied” or “Satisfied”) since we are measuring level of satisfaction. The percentages (%) do not add to 100%. All the other analysis are in the appendix.

**Table 5:** The proportions and mean satisfaction scores of respondents with the services in private facility.

Private			
Variable	Completely satisfied n (%)	Satisfied n (%)	Mean (SD)
Satisfaction with the waiting time	87 (73.1)	28 (23.5)	**4.64 (0.77)**
Regular observations during waiting time	89 (74.8)	28 (23.5)	**4.71 (0.60)**
Confidentiality of information	96 (80.7)	21 (17.6)	**4.76 (0.58)**
Privacy during vaginal examination	97 (81.5)	18 (15.1)	**4.74 (0.67)**
Privacy during delivery	80 (67.2)	30 (25.2)	**4.51 (0.87)**
Draping during delivery	77 (64.7)	36 (30.3)	**4.53 (0.81)**
Pain management during labour	36 (30.3)	55 (46.2)	**3.81 (1.18)**
Pain management after delivery	31 (26.1)	66 (55.5)	**3.89 (1.02)**
Qualified health-workers	46 (38.7)	72 (60.5)	**4.37 (0.54)**
Functional equipment	53 (44.5)	66 (55.5)	**4.45 (0.50)**
Satisfaction with response from staff	83 (69.7)	29 (24.4)	**4.58 (0.79)**
Guidance to labour companion	1 (0.8)	0 (0)	**1.90 (0.46)**
Reception of labour companion	6 (5.0)	5 (4.2)	**2.69 (1.38)**
Support and encouragement during labour	74 (62.2)	39 (32.8)	**4.50 (0.82)**
Guidance during labour	85 (71.4)	30 (25.2)	**4.62 (0.78)**
Satisfaction with delivery	89 (74.8)	23 (19.3)	**4.62 (0.83)**
Encouragement to breastfeed	84 (70.6)	26 (21.8)	**4.55 (0.84)**
Provision of information on baby’s status	61 (51.3)	56 (47.1)	**4.48 (0.61)**
Information on danger signs after delivery	50 (42)	54 (45.4)	**4.17 (0.96)**
Information on danger signs on the baby	45 (37.8)	42 (35.3)	**3.82 (1.25)**
Information on self-care	45 (37.8)	49 (41.2)	**3.96 (1.11)**
Information on care of the baby	44 (37)	50 (42)	**3.95 (1.11)**
**Overall satisfaction**	**76 (63.9)**	**41 (34.5)**	**4.60 (0.63)**

**Note:** The table only reports two levels from the Likert scale (“Completely satisfied” or “Satisfied”) since we are measuring level of satisfaction. The percentages (%) do not add to 100%. All the other analysis are in the appendix.

In terms of the mean satisfaction for individual parameters rated amongst respondents in a public facility, all were rated above 4.00 except Pain management during labour (3.81), Guidance to labour companion (1.97), and Reception of labour companion (2.50) as shown in Table 4. On the other hand, in the private facility, all were rated above 4.00 except Pain management during labour (3.81), Pain management after delivery (3.89), Guidance to labour companion (1.90), Reception of labour companion(2.69), Information on danger signs on the baby (3.82), Information on self-care 3.96), and Information on care of the baby respectively (3.95) as shown in Table 5.

### Difference in satisfaction levels between respondents in public and private facilities

The difference in the satisfaction levels of clients in private and public facilities was examined using Mann-Whitney U test and supported by questions from the FGDs. Based on the test results, there was a significant difference in the level of satisfaction in 8 out of 23 parameters as shown in Table 6.

**Table 6:** Test of significance (Mann-Whitney U test) of variation of the satisfaction level by clients in public and private facility.

	Public	Private	Man Whitney U Test	
Variable	Mean Ranks	Mean Ranks	U-Test	P- Value
Satisfaction with the waiting time	161.56	144.53	10158.50	0.068
Regular observations during waiting time	148.97	164.63	10307.50	0.103
Confidentiality of information	149.75	163.38	11248.00	0.913
Privacy during vaginal examination	155.30	154.52	11116.50	0.708
Privacy during delivery	155.99	153.42	10509.50	0.189
Draping during delivery	159.19	148.32	10469.50	0.175
Pain management during labour	154.78	155.35	11263.00	0.953
Pain management after delivery	171.70	128.34	**8132.50**	<0.001*
Qualified health-workers	158.60	149.26	10621.50	0.306
Functional equipment	166.04	137.37	**9206.50**	0.001*
Satisfaction with response from staff	161.28	143.74	**9964.50**	0.022*
Guidance to labour companion	12.06	11.18	51.50	0.737
Reception of labour companion	10.50	11.18	48.00	0.753
Support and encouragement during labour	147.31	167.29	**9843.00**	0.031*
Guidance during labour	155.00	155.00	11305.00	1.000
Satisfaction with delivery	152.77	158.56	10881.00	0.477
Encouragement to breastfeed	152.18	159.50	10769.50	0.391
Provision of information on baby’s status	160.61	146.05	10240.00	0.107
Information on danger signs after delivery	163.06	142.13	**9773.00**	0.026*
Information on danger signs on the baby	164.28	140.18	**9541.00**	0.012*
Information on self-care	167.58	134.91	**8914.00**	0.001*
Information on care of the baby	168.23	133.87	**8791.00**	<0.001*
**Overall satisfaction**	**148.22**	**165.66**	**10036.00**	**0.055**

*p-value <0.05.

The level of satisfaction amongst respondents in the public facility on pain management after delivery was statistically significantly higher than the respondents in private facilities (U = 8132.50, p<0.001).Most patients in public facility agreed that they were given pain relief medication after delivery; however, some clients in the public facilities were not given pain relief drugs during labour but were taught on conventional methods (without use of drugs) of pain relief.

“I was not given any medicine to swallow but I was told to suguamgongo (rub my back) and this helped me because it temporarily relieved the pain.” (Respondent from public facility)

On the other hand, the level of satisfaction amongst respondents in the public facility on functionality of equipment was statistically significantly higher than the respondents in private facilities (U = 9206.50, p = 0.001). Moreover, the level of satisfaction with response from staff amongst respondents in the public facility was statistically significantly higher than the respondents in private facilities (U = 9964.50, p = 0.022). Besides, results showed that the support and encouragement during labour in private facility was statistically significantly higher than in public facilities (U = 9843, p = 0.031). All the information parameters; Information on danger signs on mother after delivery(U = 9773, p = 0.026), Information on danger signs on the baby(U = 9541, p = 0.012), Information on self-care(U = 8914, p = 0.001), and Information on care of the baby(U = 8791, p<0.001), showed that the level of satisfaction was statistically significantly higher in public than in private facilities. While some clients agree that they had been provided with information, others did not remember being given any information. In public facilities, health education was offered generally in the ward before discharge while in private facility, it came out that health education which include care of baby and mother at home was offered individually on discharge once a client clears with the hospital.

“I was told that if my baby is not breastfeeding I should inform them. My baby refused to breastfeed and when I called them, they helped her and she breastfed” (respondent from public facility).

“I cannot remember being told anything” (Respondent from private facility).

“Right now we have not been given any teaching, they always give when somebody is going home.” (Respondent from private facility).

### Overall satisfaction

**Fig 1:**
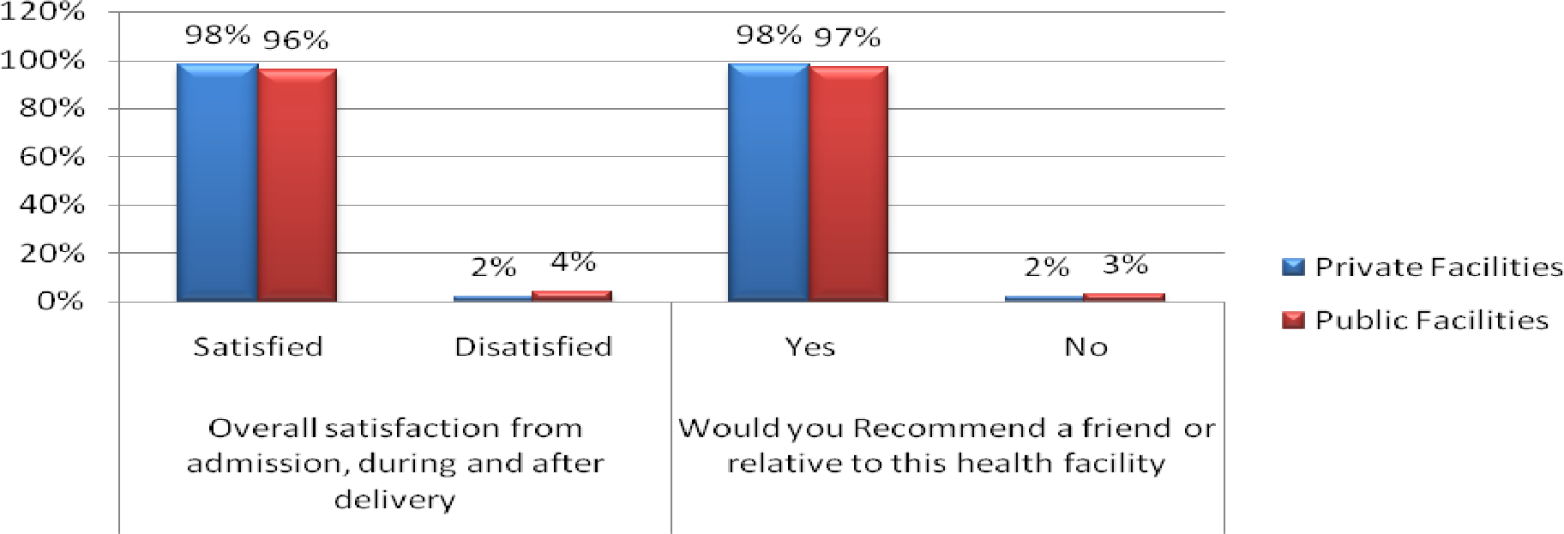
Overall satisfaction.

On the overall satisfaction with quality of services from admission, during labour, delivery and after delivery, clients from private facilities indicated higher level of satisfaction (98%) as compared to mothers from public facilities (96%) (See *Fig 1*) with no significant difference (U = 10036, p = 0.055) (See *Table 6*). It was established that almost equal proportion of respondents from private facilities (98%) and (97%) respondents from public facility would recommend a relative or friend to deliver respective health facilities (See *Fig 1*).

Some clients were happy with the way they had been treated on two different delivery occasions. However, others pointed out that despite the shortage of staff, they needed to employ someone to receive patients to the facility so that nurses can concentrate on their ward work.

“The treatment here has been good all through. This is my second delivery and I am happy, they don’t harass patients, they take you through the processes step by step. I would still recommend to others to come and deliver here.” (Respondentfrom private facility).

“Other services were okay but there are areas which need improvement for example, due to shortage of staff, sometimes there is nobody to receive the patients when they come in labourbecause the only Nurse on duty operatesbetween reception and labour ward so sometimes the patient in labour ward is left unattended. I recommend that they allocate a specific staff for reception and another specific staff for labour ward” (Respondent from public facility.)

Majority of the clients were overally happy with the positive attitude of the healthcare providers in both public and private institutions. Infact many thought that the short waiting time and availability of theatre and ambulances contributed to the improved level of satisfaction. In private facility, most clients were happy that there was no sharing of beds.

“Good service and treatment, short waiting hours, Good health education, Positive staff attitude and empathy, competent staff,availablity of theatre and ambulanceand qualified doctors and good food” (respondent from Public Facility).

“Good and empathetic staff who dont beat patients, good services and treatment,Affordable services,No sharing of beds,cleanliness of the facility and good food” (Respondent from private facility).

However, a few from the public hospital who were not satisfied mentioned sharing of beds with strangers, and rudeness of some technical and subodiante staff as some of the problems that needed resolution and were disatisfying. Whereas in private facilities, the few who were not satisfied mentioned unnecessary induction, harassment by young female staff, sharing of bathing basins and poor competence of the staff as some of the reason for dissatisfaction.

“In-adequate food, unclean toilet facilities and cold bathing water, rudeness of some technical andsurboninate staff, (Public Facility)sharing of beds with strangers and beddings are never changed-(un hygienic)” (Respondent from public facility).

“Unnnecessary induction of labour, harassment by young femalestaff, use of one basin by all patients, not being provided with pain killers during labour, poor staff competence (a patient got a perineal tear)”(Respondent from private facility).

## Discussion

The Ministry of Health (MoH)’s core values of professional practice while providing health care service in all facilities requires that there is quality and timelines [23,24]. However, the study found that there was a longer waiting time amongst the clients in the public facility than in the private facility. Moreover, despite the difference in time taken to be attended, majority of the clients from both public and private facilities reported that they were satisfied with duration of time taken to be attended. The result are similar to the finding of a study done in Kenya, Tanzania, and Ghana which found that waiting times were nearly always considerably longer at public facilities than private facilities, at least at lower level facilities [14]. Besides, the findings are consistent with those of a study conducted in Jos Metropolis of Plateau State Nigeria [3] which found that most clients in both private and public hospitals were satisfied with waiting time. The longer waiting time in the public facility could have been due to the shortage of staff and high workload [25]; however, this could be a potential area that other researchers can explore further.

This study also established that clients from both public and private facilities were satisfied in all aspects of privacy and confidentiality, during labour and delivery. These results were analogous to a study conducted by the Queensland Centre for Mothers & Babies which found that most women were satisfied with privacy in post-natal rooms in public hospital, public birth centre and private hospitals[26]. However, these findings are contrary to a study conducted in Nepal that showed that women’s satisfaction with privacy was highest in private facility whereas public hospital was rated low with respect to privacy [8].

In regard to treatment (pain management), there was no significant difference between public and private facility when it came to pain relief during labour. However, there was a significant difference in pain management after delivery with clients from public facilities more satisfied than clients from private facility. These results are different from a study conducted in Nepal which showed that there was a statistically significant difference in the combined prescription, quality, and availability of drugs between public and private hospitals where women attending private hospital had higher satisfaction level than those attending birth centre or public hospital [8]. The study results also differed with the Queensland Centre survey in that private facilities had highest level of satisfaction with care and treatment during labour, and delivery [27]. On the other hand, a study in Cambodia brought in a different perspective from this study in that clients from public facilities were not happy with treatment received in public facility as compared to private facility [6]. For instance, that study, revealed that the alleviation of pain via anesthetics during perineal suturing in public hospital would only occur if a payment was made [6]. This aspect could have been omitted in this study because the study did not interrogate the clients on pain management during any other procedure but only focused on drugs to relieve the pains during labour and after delivery. The study however found a positive outlook in the public facility which could be as a result of frequent update trainings in Maternal Neonatal care Management to public facility health workers. It was also established that majority of clients from both public and private facilities were not allowed to have labour companions, however, the percentages in public facility was slightly higher than private facility. The presence of family members is one of the key aspects that women believe constitutes good care, whether she is delivering at home or at an institution. According to traditional culture, generally a female family member, either mother or mother-in-law, accompanies the woman during child birth [28]. Similar results were found in a study conducted in Cambodia [6]. However, in Queensland Centre for Mothers & Babies, relatives were allowed to support mothers during labour and after delivery in public hospitals, public birth centers’, and private hospitals [27].

The clients from private facilities enjoyed support and encouragement by midwives during labour and delivery as opposed to the colleagues from public facility. Patient satisfaction with nursing care quality and interaction between service providers and patients are important indicators of the quality of care provided in hospitals. Two other studies from Cambodia [6] and Nepal [8] were however, dissimilar to the findings of this study. The results of this study could also be attributed to staff shortage and work overload in public facilities whereby sometimes only one staff is allocated to work both in labour ward and other areas in the maternity thus not allowing them to spend quality time with individual clients. The private facility staff on the other hand is not routinely overwhelmed and will be able to spend quality time with the clients.

On guidance during the process of delivery by midwives, the study established that clients from both public and private facilities equally agreed they were guided fully during delivery process and were encouraged to breast feed immediately thus were satisfied. Health education is an important component of maternal and child health services and Women depend on health workers to give them information on health and keep them well informed about the care they should expect. The study established a significant difference in the level of satisfaction with information provision in that a higher percentage of clients from public facilities agreed that they were provided with information on detection of danger signs in mother and in the baby after delivery, information in regard to self-care and baby care before discharge compared to their counterparts from private facility. However it is not very clear why the public facilities were more responsive in providing information as opposed to staff from private facilities. Comparable results were found in a client satisfaction survey conducted in Queensland [26] but contrary to results from a study in Jos Nigeria [3] and Nepal [8]. The study found that in overall, clients’ from both public and private facility were all satisfied with quality of child birth services from admission, during labour and delivery and they were willing to recommend the services to relatives and friends. This means that despite the quality gaps noted in specific areas of service delivery, on average, both facilities offered quality services to the clients. These results were not in concurrence with a study conducted in Kenya by Bazant & Koenig which found that dissatisfaction was greater (24%) among women who gave birth at government hospitals than (14%) at private facilities in the informal settlements [29].

The most common causes of dissatisfaction that need to be modified were long waiting time in public facility, poor pain management during labour in both public and private facilty, pain management after delivery in private facility, not allowing birth companions in both public and private facilities, lack of provision of information on detection of danger signs in baby and mother, lack of information on self care and care of baby at home in private facilities. Most mothers and babies die at home especially within the first two weeks post delivery due to infections [7] and health education on how to handle themselves and babies at home would play a major role in reducing these unnecessary deaths.

Finally, the study showed that higher numbers, (96.9%) from public and (98.1%) from private facility agreed that they would recommend the facilities to their friends and relatives. These results were slightly higher as compared to result from a study by Bazant & Koening in which approximately 60% of women giving birth at private health facilities in the slums or at government hospital responded that they would recommend the facility to others or to deliver there again [29].

### Study limitations

Conducting interviews in an area near the postnatal room or within the health facility might have encouraged some women to give accounts of care that may have been more positive than their actual experience. This could have been influenced by their colleagues who had already been interviewed and still went back to the ward or alternatively they could give positive responses due to fear of victimization by health staff even though confidentiality was assured.

This study excluded women who had undergone Caesarian Section and women with severe delivery and post-delivery complications (like women who had experienced still births and Neonatal deaths) and as such we were unable to obtain information from them. These severe complications could have been probably explained by the care rendered to these clients.

The study was conducted using a structured questionnaire and clients gave account on their experiences with child birth care services received using Likert’s scale ratings but the researcher was not able to observe processes as a verification method for the data given to assess providers' adherence to accepted standards of quality and service delivery. By directly observing the procedure, the study would have revealed more by determining the clients' experience of the client-provider interaction (qualitative phenomenological study). Despite the limitations of the study, it is likely that the findings are relevant to other women's experiences of public and private-based maternity care in Kenya and in other developing countries. There is also need to use the findings cautiously as this was a small study.

## Conclusion

This study established that there is no association between women demographic characteristics (age, marital status, religion, education level, parity, occupation, income) and choice of facility. The study further established that clients from public and private facilities were satisfied with level of privacy and confidentiality accorded to them during the childbirth services. Therefore there is no significant difference in overal clients’ satisfaction with quality of child birth services betwen public and private facilities but each facility type has its own sterngths and weaknesses in quality of different processes.

### Implications for further research

The study had no provision for establishing reasons behind long waiting hours in public facility and denial of mothers to have labour companions in the labour ward in both public and private facilities since there was no qualitative questions geared towards the same. As such there is need to investigate the reasons behind long waiting times in public facilities beyond the prescribed timeline guideline in health service charter of 2008 [23] on the time that should be taken in provision of care at the various health service delivery points. Additionally, future research could link the time taken with the period upon which the client came in and show whether emergencies may have warranted quicker attention. Also, future research could show if the stage of labour and the type of facility played a role in the differences in time taken.

The study only depended on patients experience to gauge their level of satisfaction but never used observation as means of verification whether the procedure was actually conducted to the clients’ satisfaction and as such future research should use observation but client interview should be conducted at the household level within 48 to 72 hours after delivery as this will ensure that mothers are comfortable in their own environment and more free to talk.

## Supporting information

S1 Appendix Full analysis of satisfaction scores. (PDF)

S1 File Questionnaire for the in-depth interview. (PDF)

S2 File Focused group discussion guide. (PDF)

S3 File Focused group discussion responses from facility 1. (PDF)

S4 File Focused group discussion responses from facility 2. (PDF)

S5 File Responses from the open ended questions from facility 1. (PDF)

S6 File Responses from the open ended questions from facility 2. (PDF)

S7 File Data for the study. (CSV)
