# Supplementary material for: Clients’ satisfaction with quality of childbirth services: A comparative study between public and private facilities in Limuru Sub-County, Kiambu, Kenya
Source: PLoS One. 2018 Mar 14;13(3):e0193593. doi: 10.1371/journal.pone.0193593 (PMC5851550; doi:10.1371/journal.pone.0193593)
Supplement: S1 Appendix — (PDF) [file pone.0193593.s001.pdf]

Appendix 1

Table 1: All parameters in the questionnaire

**Variable**
Level of satisfaction with the time you spent waiting for health care providers to examine you
The staff kept checking on me thus I did not feel neglected / ignored during the waiting process
Staff treated my personal information with confidence
Health facility provided privacy during vaginal examination.
During labour there were a lot of people who are non-staff around me which made me feel uncomfortable
Medical staff respected my privacy; I was not left exposed during delivery
I was given pain relief during labour as the need arose **(Pharmacological** and **Non-pharmacological)**
I was given pain relief after delivery as the need arose. **(Pharmacological)**
The health staffs in the health facility are well suited to treat mothers during labour and delivery
equipment such as blood pressure machines used on me appeared to be in good working order
How would you describe your level of satisfaction with the way staff responded to your questions and concerns during labour and delivery
My labour companion was given guidance on how best to assist me (Giving me emotional support, providing me with basic needs etc)
My labour companion was made welcome during labour and delivery process
The midwife supported and encouraged me during labour
When I had the urge to bear down, the midwife gave me clear guidance on how to do so
I was satisfied with the way the midwife conducted my delivery
I was encouraged to breastfeed my baby within one hour after delivery to foster bonding
I was informed about the health status of my baby immediately after delivery
The health staff provided me with the information on how to detect danger signs on myself after delivery
The health staff provided me with the information on how to detect danger signs in my baby
I was provided with the information on how to take care of myself at home before discharge
I was provided with the information on how to take care of the baby at home before discharge
How would you rate your level of overall satisfaction with the treatment provided by the health staff of this facility from admission, during labour, during delivery and after delivery

Table 2: The proportions and mean satisfaction scores of respondents with the OBA services in public facility

**Public**						
**Variable**	**Completely satisfied**	**satisfied**	**Not sure**	**Dissatisfied**	**Completely dissatisfied**	**Mean (SD)**
Level of satisfaction with the time you spent waiting for health care providers to examine you	120 (63.2)	59 (31.1)	0 (0)	10 (5.3)	1 (0.5)	**4.51 (0.8)**
The staff kept checking on me thus I did not feel neglected / ignored during the waiting process	127 (66.8)	52 (27.4)	1 (0.5)	7 (3.7)	3 (1.6)	**4.54 (0.82)**
Staff treated my personal information with confidence	154 (81.1)	34 (17.9)	0 (0)	2 (1.1)	0 (0)	**4.79 (0.48)**
Health facility provided privacy during vaginal examination.	158 (83.2)	26 (13.7)	1 (0.5)	5 (2.6)	0 (0)	**4.77 (0.59)**
During labour there were a lot of people who are non-staff around me which made me feel uncomfortable	140 (73.7)	41 (21.6)	4 (2.1)	4 (2.1)	1 (0.5)	**4.66 (0.68)**
Medical staff respected my privacy; I was not left exposed during delivery	137 (72.1)	45 (23.7)	0 (0)	8 (4.2)	0 (0)	**4.64 (0.7)**
I was given pain relief during labour as the need arose **(Pharmacological** and **Non-pharmacological)**	58 (30.5)	84 (44.2)	4 (2.1)	42 (22.1)	2 (1.1)	**3.81(1.13)**
I was given pain relief after delivery as the need arose. **(Pharmacological)**	103 (54.2)	65 (34.2)	1 (0.5)	20 (10.5)	1 (0.5)	**4.31 (0.96)**
The health staffs in the health facility are well suited to treat mothers during labour and delivery	89 (46.8)	93 (48.9)	2 (1.1)	3 (1.6)	3 (1.6)	**4.38 (0.74)**
equipment such as blood pressure machines used on me appeared to be in good working order	121 (63.7)	67 (35.3)	1 (0.5)	0 (0)	1 (0.5)	**4.62 (0.56)**
How would you describe your level of satisfaction with the way staff responded to your questions and concerns during labour and delivery	153 (80.5)	30 (15.8)	2 (1.1)	3 (1.6)	1 (0.5)	**4.75 (0.61)**
My labour companion was given guidance on how best to assist me (Giving me emotional support, providing me with basic needs etc)	3 (1.6)	5 (2.6)	0 (0)	0 (0)	0 (0)	**1.97 (0.3)**
My labour companion was made welcome during labour and delivery process	1 (0.5)	1 (0.5)	0 (0)	5 (2.6)	1 (0.5)	**2.5 (1.3)**
The midwife supported and encouraged me during labour	102 (53.7)	53 (27.9)	4 (2.1)	29 (15.3)	2 (1.1)	**4.18 (1.11)**
When I had the urge to bear down, the midwife gave	138 (72.6)	37 (19.5)	3 (1.6)	10 (5.3)	2 (1.1)	**4.57 (0.85)**

me clear guidance on how to do so						
I was satisfied with the way the midwife conducted my delivery	135 (71.1)	42 (22.1)	2 (1.1)	9 (4.7)	2 (1.1)	**4.57 (0.82)**
I was encouraged to breastfeed my baby within one hour after delivery to foster bonding	128 (67.4)	36 (18.9)	3 (1.6)	21 (11.1)	2 (1.1)	**4.41 (1.03)**
I was informed about the health status of my baby immediately after delivery	117 (61.6)	67 (35.3)	0 (0)	5 (2.6)	1 (0.5)	**4.55 (0.69)**
The health staff provided me with the information on how to detect danger signs on myself after delivery	108 (56.8)	60 (31.6)	2 (1.1)	18 (9.5)	2 (1.1)	**4.34 (0.97)**
The health staff provided me with the information on how to detect danger signs in my baby	109 (57.4)	32 (16.8)	2 (1.1)	45 (23.7)	2 (1.1)	**4.06 (1.28)**
I was provided with the information on how to take care of myself at home before discharge	106 (55.8)	65 (34.2)	1 (0.5)	18 (9.5)	0 (0)	**4.36 (0.9)**
I was provided with the information on how to take care of the baby at home before discharge	106 (55.8)	66 (34.7)	1 (0.5)	17 (8.9)	0 (0)	**4.37 (0.89)**
How would you rate your level of overall satisfaction with the treatment provided by the health staff of this facility from admission, during labour, during delivery and after delivery	101 (53.2)	82 (43.2)	1 (0.5)	5 (2.6)	1 (0.5)	**4.46 (0.7)**

Table 3: The proportions and mean satisfaction scores of respondents with the OBA services in private facility

Private						
**Variable**	**Completely satisfied**	**satisfied**	**Not sure**	**Dissatisfied**	**Completely dissatisfied**	**Mean (SD)**
Level of satisfaction with the time you spent waiting for health care providers to examine you	87 (73.1)	28 (23.5)	0 (0)	1 (0.8)	3 (2.5)	**4.64 (0.77)**
The staff kept checking on me thus I did not feel neglected / ignored during the waiting process	89 (74.8)	28 (23.5)	0 (0)	1 (0.8)	1 (0.8)	**4.71 (0.6)**
Staff treated my personal information with confidence	96 (80.7)	21 (17.6)	0 (0)	1 (0.8)	1 (0.8)	**4.76 (0.58)**
Health facility provided privacy during vaginal examination.	97 (81.5)	18 (15.1)	0 (0)	3 (2.5)	1 (0.8)	**4.74 (0.67)**
During labour there were a lot of people who are non-staff around me which made me feel uncomfortable	80 (67.2)	30 (25.2)	0 (0)	8 (6.7)	1 (0.8)	**4.51 (0.87)**
Medical staff respected my privacy; I was not left exposed during delivery	77 (64.7)	36 (30.3)	0 (0)	4 (3.4)	2 (1.7)	**4.53 (0.81)**
I was given pain relief during labour as the need arose **(Pharmacological** and **Non-pharmacological)**	36 (30.3)	55 (46.2)	2 (1.7)	22 (18.5)	3 (2.5)	**3.81 (1.18)**
I was given pain relief after delivery as the need arose. **(Pharmacological)**	31 (26.1)	66 (55.5)	2 (1.7)	18 (15.1)	2 (1.7)	**3.89 (1.02)**
The health staffs in the health facility are well suited to treat mothers during labour and delivery	46 (38.7)	72 (60.5)	0 (0)	1 (0.8)	0 (0)	**4.37 (0.54)**
Equipment such as blood pressure machines used on me appeared to be in good working order	53 (44.5)	66 (55.5)	0 (0)	0 (0)	0 (0)	**4.45 (0.5)**
How would you describe your level of satisfaction with the way staff responded to your questions and concerns during labour and delivery	83 (69.7)	29 (24.4)	1 (0.8)	5 (4.2)	1 (0.8)	**4.58 (0.79)**
My labour companion was given guidance on how best to assist me (Giving me emotional support, providing me with basic needs etc)	1 (0.8)	0 (0)	0 (0)	104 (87.4)	13 (10.9)	**1.90 (0.46)**
My labour companion was made welcome during labour and delivery process	6 (5.0)	5 (4.2)	0 (0)	3 (2.5)	0 (0)	**2.69 (1.38)**
The midwife supported and encouraged me during	74 (62.2)	39 (32.8)	0 (0)	5 (4.2)	1 (0.8)	**4.50 (0.822)**

labour						
When I had the urge to bear down, the midwife gave me clear guidance on how to do so	85 (71.4)	30 (25.2)	0 (0)	2 (1.7)	1 (0.8)	**4.62 (0.78)**
I was satisfied with the way the midwife conducted my delivery	89 (74.8)	23 (19.3)	2 (1.7)	3 (2.5)	1 (0.8)	**4.62 (0.83)**
I was encouraged to breastfeed my baby within one hour after delivery to foster bonding	84 (70.6)	26 (21.8)	0 (0)	9 (7.6)	0 (0)	**4.55 (0.84)**
I was informed about the health status of my baby immediately after delivery	61 (51.3)	56 (47.1)	1 (0.8)	0 (0)	1 (0.8)	**4.48 (0.61)**
The health staff provided me with the information on how to detect danger signs on myself after delivery	50 (42)	54 (45.4)	3 (0.8)	13 (10.9)	1 (0.8)	**4.17 (0.96)**
The health staff provided me with the information on how to detect danger signs in my baby	45 (37.8)	42 (35.3)	0 (0)	29 (24.4)	3 (2.5)	**3.82 (1.25)**
I was provided with the information on how to take care of myself at home before discharge	45 (37.8)	49 (41.2)	1 (0.8)	23 (19.3)	1 (0.8)	**3.96 (1.11)**
I was provided with the information on how to take care of the baby at home before discharge	44 (37)	50 (42)	3 (0.8)	23 (19.3)	1 (0.8)	**3.95 (1.11)**
How would you rate your level of overall satisfaction with the treatment provided by the health staff of this facility from admission, during labour, during delivery and after delivery	76 (63.9)	41 (34.5)	0 (0)	1 (0.8)	1 (0.8)	**4.60 (0.63)**
