# Supplementary material for: Clients’ satisfaction with quality of childbirth services: A comparative study between public and private facilities in Limuru Sub-County, Kiambu, Kenya
Source: PLoS One. 2018 Mar 14;13(3):e0193593. doi: 10.1371/journal.pone.0193593 (PMC5851550; doi:10.1371/journal.pone.0193593)
Supplement: S1 File — (PDF) [file pone.0193593.s002.pdf]

CLIENTS'SATISFACTION WITH QUALITY OF CHILDBIRTH SERVICES. "A COMPARATIVE STUDY BETWEEN PUBLIC AND PRIVATE FACILITIES IN LIMURU SUB-COUNTY-KIAMBU –KENYA.

Questionnaire Source: The questionnaire has been adapted from : Mfundisi N.P**,** Melese T, Gebrehiwot Y, Bisetegne ,D,Habte D.(2014)**,** Karkee R, Lee A.H& Pokharel P.K.(2014.)

This Questionnaire will be used to establish Patient satisfaction with healthcare during childbirth

PATIENT SATISFACTION WITH HEALTH SERVICES DURING LABOUR, DELIVERY AND AFTER DELIVERY

Rating scale: **5**= Completely Satisfied/ Agree; **4**= Satisfied/Agree; **3**= Not Sure; **2**=Dissatisfied /Disagree; **1**= Completely Dissatisfied/Disagree

Facility Code_______________________

Date	Patient code

SECTION A –General characteristics

Demographic Data

Kindly provide the following demographic information about yourself/

Please tick the appropriate circle

01. How old are you? Indicate Age in completed years: ______________________

02. What is your marital status?

Single (**1)** Married monogamous **(2)** Married polygamous **(3**) Divorced (**4**) Widowed (**5**)

03. What is your religion?

Christian protestant **(1**) Christian catholic **(2)** Muslim **(3)** No religion **(4)** Others Specify________ **(5)**

Socio-Economic

04. What is the highest level of education that you attained?

No education (Never been to school) (**1**) Primary (**2**) Secondary (**3)** College **(4)** University **(5)**

05. What is your occupation?

Student (**1**) Unemployed (**2**) Self Employed **(3)** Formal Employment/salaried (**4**)

06. How much is your income per month (approximate in Ksh.)

None **(1**) 1 – 5,000 **(2**) 5,001 - 10,000 **(3)** above 10,000 **(4)**

Obstetric data:

07. What is your **parity** ?(How many times have your pregnancy ended in a live birth and how many times have you miscarried or had an abortion?)

First pregnancy (Primigravida) (**1**) Para 2-5) (**2**) Para 5 and above (**3**)

Patient satisfaction with health services during childbirth

Kindly respond by indicating the level to which you are in agreement/satisfied with the statements provided.

SECTION B. Client Satisfaction with turnaround time (Time taken from arrival in facility to time attended by health provider)

08. How long did you wait between the time you first arrived at this clinic and the time health provider attended to you for consultation? Indicate actual waiting time in **hours:** ____________________________

0-30 mins **(1)** 30 mins – 1 hour (**2**) 1hr- 2hrs (**3)** Over 2 hrs (**4**)

**S/No**	**CODE**	**5**	**4**	**3**	**2**	**1**
	**Statement**	**Completely satisfied**	**Satisfied**	**Not sure**	**Dissatisfied**	**Completely Dissatisfied**
09	On a scale of one to five how would you describe your level of satisfaction with the time you spent waiting for health care providers to examine you?					
10	To what extent do you agree with this statement? The staff kept checking on me thus I did not feel neglected / ignored during the waiting process.					

Section C. Clients satisfaction with privacy and confidentiality

	**Statement**	**Completely satisfied**	**Satisfied**	**Not sure**	**Dissatisfied**	**Completely Dissatisfied**
11	**How would you rate your level of agreement with the following statement?**<br><br>The staff treated my personal information with confidence					

	Statement	Completely satisfied	Satisfied	Not sure	Dissatisfied	Completely Dissatisfied
12	**To what extent do you agree with the following statement?**<br><br>The health facility provided privacy during vaginal examination.					
13	During labour there were a lot of people who are non-staff around me which made me feel uncomfortable					
14	The medical staff respected my privacy; I was not left exposed during delivery (2nd Stage).					
**Section D. Satisfaction With Treatment During Labour and after Delivery**						
15	**How would you describe your level of agreement with pain management during labour?**<br><br>I was given pain relief during labour as the need arose ***(Pharmacological*** *and* ***Non-pharmacological)***					
16	**How would you describe your level of agreement with pain management after you delivered your baby?**<br><br>I was given pain relief after delivery as the need arose. ***(Pharmacological)***					
17	**To what extent do you agree with the following statement**					

	Statement	Completely satisfied	Satisfied	Not sure	Dissatisfied	Completely Dissatisfied
	The health staffs in the health facility are well suited to treat mothers during labour and delivery					
18	The equipment such as blood pressure machines used on me appeared to be in good working order					
19	How would you describe your level of satisfaction with the way staff responded to your questions and concerns during labour and delivery					
20	I was allowed to have a birth companion<br><br>*If **Yes** go to **No. 22***<br><br>*If **No** skip to **No. 23***	**Yes (1)**	**No (2)**			
	**What is your level of agreement with the following statement**					
21	My labour companion was given guidance on how best to assist me (Giving me emotional support, providing me with basic needs etc)					
22	My labour companion was made welcome during labour and delivery process,					
23	The midwife supported and encouraged me during labour					

	Statement	Completely satisfied	Satisfied	Not sure	Dissatisfied	Completely Dissatisfied
24	When I had the urge to bear down, the midwife gave me clear guidance on how to do so					
25	I was satisfied with the way the midwife conducted my delivery					
26	I was encouraged to breastfeed my baby within one hour after delivery to foster bonding					
**Section E: Satisfaction With Information after Delivery and Before Discharge**						
	**To what extent do you agree with the following statement?**					
27	I was informed about the health status of my baby immediately after delivery					
28	The health staff provided me with the information on how to detect danger signs on myself after delivery.					
29	The health staff provided me with the information on how to detect danger signs in my baby					
30	I was provided with the information on how to take care of myself at home before discharge					
31	I was provided with the information on how to take care of the baby at home before discharge					
32	How would you rate your level of overall satisfaction with the treatment provided by the health staff of this facility from					

	Statement	Completely satisfied	Satisfied	Not sure	Dissatisfied	Completely Dissatisfied
	admission, during labour, during delivery and after delivery?					

Did you have any positive feedback that you would like to share regarding your delivery?

____
____
____
____
____
____

Did you have any negative feedback that you would like to share regarding your delivery?

____
____
____
____
____
____

Would you recommend for a relative or friend to come and deliver in this health facility?

(1) Yes

(2) No

Kindly explain the reason to your response

____

Thank You for dedicating your time to answer my questions.
