# Supplementary material for: Clients’ satisfaction with quality of childbirth services: A comparative study between public and private facilities in Limuru Sub-County, Kiambu, Kenya
Source: PLoS One. 2018 Mar 14;13(3):e0193593. doi: 10.1371/journal.pone.0193593 (PMC5851550; doi:10.1371/journal.pone.0193593)
Supplement: S2 File — (PDF) [file pone.0193593.s003.pdf]

FOCUSED GROUP DISCUSSION GUIDE

Interview Code---------------------------

Venue---------------------------------------

Introduction

Prayer

Consent

Theme 1.Satisfaction with -turn around time

Sub-theme 1. Tell me how long you waited from the time you reached the facility until you were attended by the health staff, examined and admitted? *Probe.*

Sub theme 2.What is your feeling concerning the duration of time spent waiting to be attended by the health staff about this waiting? *Probe was you satisfied? If not what recommendation would you make for future improvement?*

Theme 2.Satisfaction with privacy and confidentiality

Subtheme 1.Kindly shares with me your experience concerning privacy during vaginal examination, during labour and during delivery? *Probe*

Subtheme 2.How would you describe level of satisfaction with privacy accorded during delivery? Were you comfortable with the privacy accorded during delivery? *Probe .If not what would you like to be done to improve privacy to clients*

Sub theme 3.Do you feel that the staff handled your personal information in confidence? P*robe why for the answer provided .If not why? What suggestions do you have for improvement?*

T**heme 3 Satisfaction with Treatment/Emotional support during labour and delivery**

Subtheme 1 .Were you given any pain relief drug during labour?

Subtheme 2. How would you describe the nurses responses to your concerns and worries during labour?

Subtheme 3. How would you describe the type of care and support you received support received from the midwives during labour and delivery

Subtheme 4 Were you allowed to have a birth companion during labour and delivery? *Please Explain further*

Theme 4.Information after delivery and before discharge

Were told the signs you should look out for in a baby to detect that your baby's health is in danger?

Please tell me about the information you were given concerning yourself care and care of baby after delivery and before going home?

Theme 5, Overall satisfaction with care on admission, during labour and delivery

How would you describe the way hospital staff looked after you on admission, during labour and after your baby was born?

Thank you for your cooperation
