# Supplementary material for: Clients’ satisfaction with quality of childbirth services: A comparative study between public and private facilities in Limuru Sub-County, Kiambu, Kenya
Source: PLoS One. 2018 Mar 14;13(3):e0193593. doi: 10.1371/journal.pone.0193593 (PMC5851550; doi:10.1371/journal.pone.0193593)
Supplement: S3 File — (PDF) [file pone.0193593.s004.pdf]

TIGONI SUB-COUNTY HOSPITAL L001

FOCUSED GROUP DISCUSSION RESPONSES

8 RESPONDENTS

Resp1,Resp 2,Resp 3,Resp 4,Resp 5,Resp 6,Resp 7,Resp 8

Question 1.What can you say about the time you took to before being attended?

Respondent 1.I took a short time,they did very fast.Was taken to labour ward and examined very fast.

Q 2.What can you say about your privacy and confidentiality when being examined

Resp 1.I was alone in labour ward and it was very confidential.After being examined,I was told in details how far she was and what to expect.I was well treated.

Q3.What was your experience with treatment during labour?

Resp.5-I was in the ward, relatives were allowed in and I preferred when there are people around unlike ukiwa peke yako because some people are first time parents and things may go wrong.

Q4.During labour were you given any drug or advised on what to do to reduce pain?

Resp 1.I was told to "sugua mgongo"-rub my back)and this helped in reducing pain.

Q5.What can you comment on privacy during the time you were delivering?

Resp 1.I was well received and after being examined,I was told my baby was lying in breech position and during delivery I was only alone with the Nurses.

Q6.After delivery were you given any drug to reduce pain?

All respondents-Yes,We were given panadols

Q7.During delivery who assisted you?

Resp 3.I had breech delivery and the baby had cord around the neck but the Nurses were of great help.

Q.8. Was the birth companion allowed to stay with you during the delivery process?

All Respondents.None of us was allowed to have a birth companion

Q9.What can you comment on the experience and skills of the staff that handled you?

Resp.7. Very good,the Nurses have been very nice and kind.None has been mean.

Q10.What health education were you given on care for yourself or the baby before discharge?

I was told to go back to clinic after 2 weeks for myself and also to take the baby back to clinic at six weeks and also to start Family Planning

Q11.What about the care of the baby?

Resp.6. We have not been told anything on care of the baby.They always tell people after immunizing the baby.

Resp 4.We have not been given any health education because today is a weekend and education is only given when the babies are being immunized and today it's not a vaccination day.Otherwise the treatment has been good all through .This is my second delivery here .They don't harakisha watu they do the procedures step by step.I will advice the others to come here.

Q12.How would you rate the overall satisfaction with the treatment received from admission, during delivery until discharge?

Rep 1.I was satisfied and would recommend to others to come here.

Resp 3. Was taken care of well all through

Resp 8.I was not very satisfied because I was left alone in labour ward for almost two hours

Resp 4.I was in labour from 8.00 am to 3.00pm and when the Mzungu came, the delivery happened.

All Respondents-When there is a Mzungu,then delivery happens

Resp.7.I was satisfied. My first delivery was Caeserian Section but this time, the Mzungu made me deliver normally.

Q12.Do you have any other comment?

Rep 1.Food here is very little

Resp 2.We share beds here with strangers

Resp 4.The food is not balanced

Resp 5.We bathe with cold water after delivery

Resp.6.There are no blankets and this place is very cold.

Resp 8. The staff are okay but *vifaa vya kutumia ndio hawajapewa.*

Resp.7. Delivery is free but the facility is not upto standard, we are not provided with pads.
