# Supplementary material for: Clients’ satisfaction with quality of childbirth services: A comparative study between public and private facilities in Limuru Sub-County, Kiambu, Kenya
Source: PLoS One. 2018 Mar 14;13(3):e0193593. doi: 10.1371/journal.pone.0193593 (PMC5851550; doi:10.1371/journal.pone.0193593)
Supplement: S4 File — (PDF) [file pone.0193593.s005.pdf]

LIMURU NURSING HOME L002

FOCUSED GROUP DISCUSSION 23-5-2015

Respondents

Resp 1,Resp 2,Resp 3,Resp 4,Resp 5,Resp 6,Resp 7,Resp 8

Q1.What can you comment on the time you spent before getting attended?

All Respondents-I was attended to immediately and was satisfied

Q2.How can you rate the level of satisfaction with the privacy during Vaginal examination?

All Respondents. There was no problem.Every body was being examined when she is alone with the Nurses.

Q3.What can you comment on level of privacy during delivery?

Resp.3.I was alone with the Nurse and there was nobody else in the room.

Q4.What can you comment on confidentiality?

Resp 4. History taking was done in confidence and nobody else heard what I was being asked.

Q5.Were you given any pain reliever during labour or told of anything you need to do in order to relieve pain?

All respondents-We were not given any pain reliever

Q6.How can you rate the level of satisfaction with the Nurses responses to your questions?

Resp 4.The Nurse talked to us well and answered our questions

Q7.What is the level of satisfaction with the support given during labour and delivery?

Resp 1. I was not given any support during labour because I gave birth immediately.

Resp.5. I gave birth immediately

Resp.3. Got Support, delivered almost immediately, Nurse talked to me.

Resp.2. Got Support,Nurse talked to me well and reassured me.

Resp.4 Got support, Nurse talked to me.

Resp.6.Got a lot of support from nurses,I was reassured and was satisfied

Q8.What is the level of satisfaction with support during delivery

R2.I was supported by three nurses and I delivered well.

Resp 1,R5,R4&R3..I got a lot of support from the Nurse.

Q9.What is your comment on health education provided on self care and care of the baby before discharge.

Resp 6.Niliambiwa ati mtoto akilia sana niambie daktari."*I was told that when the baby cries a lot I should tell the Doctor"*

I was also told that when I bleed a lot I should inform the Doctor.

Resp 1.I was told that if the baby is not breastfeeding I should call the nurse.

Resp 6.My cbaby was not breastfeeding and when I called the Nurse,she came helped me and the baby breastfed

Q11.What is your comment on health education provided on care of baby after discharge?

All Respondents-No health education yet because this is done individually after clearing the hospital bill and ready to go home

Q12.What is your overall satisfaction wih care accorded from admission,during labour,delivery until discharge?

Resp 6-I have not seen any problem and can recommend this facility to other patients.

Resp.1-R1,R5,R4,R9-The services are good,the sheets are changed everyday,Nurses help you with the baby if you can not walk.

Do you have any other comment?

Resp.6,1,7,3,4 The services are perfect,there is no improvement neded
