# Supplementary material for: Clients’ satisfaction with quality of childbirth services: A comparative study between public and private facilities in Limuru Sub-County, Kiambu, Kenya
Source: PLoS One. 2018 Mar 14;13(3):e0193593. doi: 10.1371/journal.pone.0193593 (PMC5851550; doi:10.1371/journal.pone.0193593)
Supplement: S5 File — (PDF) [file pone.0193593.s006.pdf]

OPEN ENDED QUESTIONS

RESPONSES

CLIENTS SATISFACTION WITH CHILD BIRTH SERVICES "A COMPARISON BETWEEN PUBLIC AND PRIVATE FACILITIES IN KIAMBU COUNTY"

FACILITY CODE T001

**Respondent**	***Did you have any positive feedback you would like to share regarding your delivery?*** ***Did you have any positive feedback you would like to share regarding your delivery?***	***Did you have any negative feedback you would like to share regarding your delivery?*** N	***Would you recommend for a relative/friend to deliver in the facility. If YES Reason?***	***Would you recommend for a relative/friend to deliver in the facility .If NO Reason?***
**R1**	I was happy with the way Nurses treated me during delivery They instructed me on what to do. For example to breathe through the mouth when experiencing pain	The food served after delivery was not enough .For example food is served once a day and the food is very little. ***Recc***. Pregnant mothers should be fed well	The treatment is good. The staff are constantly checking on us and are concerned with our safety	
**R2**	The Nurses were good and were concerned to see how we are fairing on with our babies		The Nurses are concerned with our welfare	
**R3**	The staff really took good care of me .I thank them	If you don't follow the instructions given by the Nurses, they shout at you	The staff are very competent in their work, they are disciplined and have good rapport. We are not left hungry	
**R4**	Nurses are very kind .They offer to take care of us despite high workload	There is no oxygen in labour ward for example one mother had no energy to push so instead of oxygen some Nurses pressed her abdomen while others were guiding the baby Even my other child I delivered here and I have come again because I like the way Even my other child I delivered here and I have come again because I like the way to come out	I like this place because even the other child I delivered here and I have come again because I like the treatment here.	

Respondent	*Did you have any positive feedback you would like to share regarding your delivery?*<br>*Did you have any positive feedback you would like to share regarding your delivery?*	*Did you have any negative feedback you would like to share regarding your delivery?*<br>N	*Would you recommend for a relative/friend to deliver in the facility. If YES Reason?*	*Would you recommend for a relative/friend to deliver in the facility .If NO Reason?*
		**RECC.**Need for oxygen in Labour ward		
R5	The staff aregood ,they take care of patients well,and patients are not neglected at all.		Because it is a good facility ,staff are good ,patients are not neglected ,the leave the patient when she is very comfortable.	
R6	Facility provides good services<br>The staff do not ignore patients, they are attended to when need arises<br>Staff educate mothers on how to take care of their babies at home	Bedding is not changed sometimes and sometimes patients are not given blankets due to shortage	They will be taken care of well<br>Being a government facility, it is affordable<br>Services are free	
R7	I was attended to immediately<br>Nurses do not abuse and shout at clients	There was no privacy in the delivery room, there was two of us in the same delivery room, and I did not like the way I was exposed	The staff treat people well and do not shout at clients	
R8	Staff are good in offering services they have good interaction with patients,They should continue with the same spirit of helping mothers.		The facility is good.<br>Staff have good communication skills and provide good services.	
R9	The staff handled me well, they encouraged me especially when I lost energy while pushing. The midwife was really there for me and I appreciate	When I was having pain during labour,at some point the midwife seemed not to care,		Because I was properly handled,was satisfied with the way I was treated,Health education that the staff offer is very good because the mother has confidence in taking care of the baby

Respondent	***Did you have any positive feedback you would like to share regarding your delivery?***<br>***Did you have any positive feedback you would like to share regarding your delivery?***	***Did you have any negative feedback you would like to share regarding your delivery?***<br>N	***Would you recommend for a relative/friend to deliver in the facility. If YES Reason?***	***Would you recommend for a relative/friend to deliver in the facility .If NO Reason?***
				when she goes home.
R10	They have good hygiene practices.<br>The staff treat patients well.	The toilets are dirty.<br>The food is poorly prepared.	I was happy with the way I was treated.	
R11	I did not wait for long before being attended to.	Sharing of beds.<br>Toilets are dirty.	The staff attend to clients well and are concerned about their patients.	
R12	The staff are supportive to clients.		The hospital provided the best maternity care.	
R13	The staff attend to patients well and they help in case of any complications during labour.<br>The nurses gave me health education on taking care of the baby.	There are fewer beds and when there are many patients then we have to share.<br>The facility does not provide warm water for shower.	The staff are well skilled and take care of patients well.	
R14	Staff take good care of patients.	Sometimes the nurses take long to respond when called to help.	Patients are well taken care of.	
R15	Staff offer good services to patients and interact well with us.	The bedding and toilets are not clean	The services offered are free and staff are concerned with the patients.	
R16	Staff are concerned about the well being of patients and keep on checking on us.	I was not given a pain reliever when the pain was too much as I was in labour.	Staff offer good services.<br>The maternity services are free.	
R17	The staff are not rude	The toilets and the wards are dirty.<br>The bedding is not changed.<br>There are fewer beds and therefore patients are forced to share	The staff provide good service.	
R18	Staffs are well skilled in offering delivery services.<br>The staff offer health talks to mothers.	It took long before I was attended to.<br>I was asked to go to Limuru Nursing Home	They offer good services.	

Respondent	***Did you have any positive feedback you would like to share regarding your delivery?*** ***Did you have any positive feedback you would like to share regarding your delivery?***	***Did you have any negative feedback you would like to share regarding your delivery?*** N	***Would you recommend for a relative/friend to deliver in the facility. If YES Reason?***	***Would you recommend for a relative/friend to deliver in the facility .If NO Reason?***
		after a scan showed a breech presentation.		
R19	I did not wait long before being attended to.<br>I was well attended to throughout the labor process		They offer good services.<br>Patients do not wait for long before being attended to.	
R20	The staff are friendly and ready to help.		The facility is good and they offer good services.	
R21	The staff offer good services.<br>The wards are clean.		The facility offers good services.<br>The wards are clean.	
R22	I was happy with the services offered.		The facility offers very good services.	
R23	I was happy with the way I was treated.		The facility is good, patients are well treated.	
R24	I was happy with the way I was delivered and how they took care of the baby in the New Born Unit.		It is a good facility and patients are well treated.	
R25	I did not wait for long before being attended,The Nurses really supported me well during labour and delivery and answered my questions well,	The health facility doesn't have a scanning machine one has to go somewhere else and its expensive to do it in private hospital	Yes<br>People don't wait for long before being attended and being a public hospital, the services are free.	
R26	Whenever I needed help, Nurses were ready to help me ,I appreciate the health education I was given and also happy with the Nurses, They were concerned about me.		Yes<br>Because the patients don't wait for long before being attended to .The staff provide HIV services to the mothers and they are counselled on how to take care of their babies.	
R27	I didn't take long before being attended,I was happy with the		Yes	

Respondent	***Did you have any positive feedback you would like to share regarding your delivery?***<br>***Did you have any positive feedback you would like to share regarding your delivery?***	***Did you have any negative feedback you would like to share regarding your delivery?***<br>N	***Would you recommend for a relative/friend to deliver in the facility. If YES Reason?***	***Would you recommend for a relative/friend to deliver in the facility .If NO Reason?***
	Nurse who conducted my delivery,she is very good.The Nurses Kept on checking on me during the labor process.		Because I was treated well and given good services	
R28	I did not take long before being attended and when I felt like bearing down, the Nurse was there to help and encourage me.		Yes<br>Because they offer good services and patients don't wait for long.	
R29	I didn't not wait for long before being attended ,I was happy with the way staff took care of me, They responded to my needs whenever I called them.		Yes<br>The Nurses have good customer care; they kept on checking on me and were answering my questions.	
R30	I was happy because when the Nurses realized that my baby was having breech presentation ,they took me to theatre but fortunately I delivered normally while still in theatre		Yes<br>Because the services are good. It is a good facility	
R31	I liked the way the Nurse supported me during delivery being my first baby.I did not feel neglected.The staff are friendly.		Yes<br>Because the services are free,staff are ready to offer services despite high workload,they are ambulance services and a thetre in case of complications.	
R32	I was attended to immediately I arrived .The Nurses are vey compassionate and are willing to help incase of any problem	.The beds are not enough And people are made to share beds yet some patients don't want to share, I don't like the issue of sharing beds	Yes<br>Because the Nurses and Doctors are very good,They attend to patients immediately.	
R33	The Nurses are good .They worked as a team to assist me deliver when I had no energy, The food is good and they offer good services			

Respondent	***Did you have any positive feedback you would like to share regarding your delivery?***<br>***Did you have any positive feedback you would like to share regarding your delivery?***	***Did you have any negative feedback you would like to share regarding your delivery?***<br>N	***Would you recommend for a relative/friend to deliver in the facility. If YES Reason?***	***Would you recommend for a relative/friend to deliver in the facility .If NO Reason?***
R34	I was attended to in less than five minutes, The staff are really good, they supported me throughout my labour process		Yes<br>Because they offer good services and don't shout at patients	
R35	The staff supported me in every stage of my labour and delivery and was happy with the services		Yes<br>I was also referred here by a friend who liked the services and I have also liked the services	
R36	When the Nurses realized that I was not progressing well, they augmented the labour,kept on checking on me and I deliverd a healthy baby,I appreciate the health education offered to me on labour pain management and care of the baby at home.		Some mothers were really in a lot of pain and kept on making a lot of noise and when they called on the Nurse she never came to help them out.	
R37	Nurses are friendly,they provide good ser vices and and have no discrimination on patients		Yes<br>The facility is good, clean and Nurses and Doctors work in Unity	
R38	The facility is clean, services are are good, and the food is good	Sometimes when patients call the Nurses they are ignored and some staff are rude	Yes<br>Because the place is clean, services are good and are free	
R39	I was attended to immediately and served well by Nurses .They are attentive to patients problems		Yes<br>It is agood facility and the staff attend to patients immediately they arrive.	
R40	The Nurses didn't shout at me when I delivered at the door as I was going to labor ward			
R41	The Nurses provide great services ,I did not wait for long before being attended		Yes, Because the staff really take good care of patients you don't wait for long before being attended to	
R42	The ward is clean	The Nurses ignored me whenever I needed help,I	Yes	

Respondent	***Did you have any positive feedback you would like to share regarding your delivery?***<br>***Did you have any positive feedback you would like to share regarding your delivery?***	***Did you have any negative feedback you would like to share regarding your delivery?***<br>N	***Would you recommend for a relative/friend to deliver in the facility. If YES Reason?***	***Would you recommend for a relative/friend to deliver in the facility .If NO Reason?***
		went to the Nurses desk and they told me to go back to the ward,that I still have time I stop disturbing them,when I felt like bearing down,I went to labour ward and started pushing and when my baby was out is when the Nurses came up to help me	When you get a good Nurse services are good	
R43	They provide good services	The toilets are not very clean	Yes<br>The staff are kind, they feed mothers well, they take care of babies well, keep on checking on patients	
R44	Patients are friendly,Nurses have good communication skills ,Nurses keep on checking on patients ,cleanliness in the ward	The surbodinate staffs especially those who serve food talk rudely to patients.	Yes<br>The staff are good and really support patients and patients are not ignored, there are enough Doctors	
R45	I was not made to stay for long because I was a transfer from another hospital and immediately I reached I was attended and given good support		Yes<br>I was very happy ,I was treated as a human being not like where I was before	
R46	The services are good I was treated well, was given good instructions especially during labor and given good food		Yes<br>Because the services provided are good and when they come they will be treated well	
R47	They really helped my baby because when they realized that there was meconium they immediately put a line on me and this helped my baby not to get tired was very happy with the services provided.		Because I have been well taken care and they too will be well taken care of.	

Respondent	***Did you have any positive feedback you would like to share regarding your delivery?***<br>***Did you have any positive feedback you would like to share regarding your delivery?***	***Did you have any negative feedback you would like to share regarding your delivery?***<br>N	***Would you recommend for a relative/friend to deliver in the facility. If YES Reason?***	***Would you recommend for a relative/friend to deliver in the facility .If NO Reason?***
R48	The Nurses treated me well	I did not like the fact that we were shared the bed with another mother	Yes<br>The services are good except for sharing of beds	
R49	I liked the services I got	I did not like the fact that we shared a bed with another mother		No, Because the beds are not enough and they will be made to share the beds.
R50	The services I got were good		Yes, The services are good .I like how I was treated	
R51		The food is not good, When one lives far from the hospital,they may not get people to bring her . water for showering is too cold<br>I was also uncomfortable sharing a bed with another mother.	Yes<br>The services are good except the bad food and cold water	
R52	I liked how I was treated by the Nurses		Yes<br>The treatment I got was good and would recommend the facility for a relative	
R53	The services are good		Yes<br>The treatment is good except for issue of sharing beds	
R54	The Nurses treated me well and the services I got were good		Yes<br>The services I got were good	
R55	The services are good	The sharing of beds is not good	Yes because the Nurses are good and the place is also good	
R56		The service wasn’t good.The time I was in labour I was beaten up.I was alone during labour		No<br>The service wasn’t that good. Although Some Nurses are

Respondent	***Did you have any positive feedback you would like to share regarding your delivery?***<br>***Did you have any positive feedback you would like to share regarding your delivery?***	***Did you have any negative feedback you would like to share regarding your delivery?***<br>N	***Would you recommend for a relative/friend to deliver in the facility. If YES Reason?***	***Would you recommend for a relative/friend to deliver in the facility .If NO Reason?***
		and nobody encouraged me.		good,I did not like the services I Received
R57	They provide good services especially lab our ward .I was happy with the Nurse who conducted my delivery		Yes, Because they provide good services	
R58	I was happy with the Nurse who conducted my delivery.He really supported and encouraged me .The Nurses are good and cooperative and the facility is clean,especially the ward.		Yes<br>Because the facility is good, well equipped ,has theatre ,incase of any emergency somebody will not be referred but taken to theatre and well taken care of.	
R59	The Nurses are good and assisted me well during delivery. The ward is clean, casuals are good and not rude to patients	The watchman needs to change .He did not treat me well. He does not talk to visitors	Yes ,because the staff are good ,well trained ,hospital well equipped.	
