# Supplementary material for: Clients’ satisfaction with quality of childbirth services: A comparative study between public and private facilities in Limuru Sub-County, Kiambu, Kenya
Source: PLoS One. 2018 Mar 14;13(3):e0193593. doi: 10.1371/journal.pone.0193593 (PMC5851550; doi:10.1371/journal.pone.0193593)
Supplement: S6 File — (PDF) [file pone.0193593.s007.pdf]

OPEN ENDED QUESTIONS

RESPONSES

CLIENTS SATISFACTION WITH CHILD BIRTH SERVICES "A COMPARISON BETWEEN PUBLIC AND PRIVATE FACILITIES IN KIAMBU COUNTY"

FACILITY CODE L002

Respondent	*Did you have any positive feedback you would like to share regarding your delivery?*<br>*Did you have any positive feedback you would like to share regarding your delivery?*	*Did you have any negative feedback you would like to share regarding your delivery?*	*Would you recommend for a relative/friend to deliver in the facility. If YES Reason?*	*Would you recommend for a relative/friend to deliver in the facility .If NO Reason?*
**R51**	The facility staff treat patients well .Patients don't wait for long before being attended	none	Yes<br>Patients are treated well. Staff are every good ,caring and understanding. The facility is clean, we get enough food	.
**R52**	The facilty provides very good services .Nurses are caring.Keep it up		Yes, The staff are caring, food is good, very good services ,clean facility and it's not expensive	
**R53**	The place is very clean and the food is good. The Nurse who was helping me to deliver was vey good, instructed me well on what to do,the services were very good	I was not happy with the way a Nurse slapped that mother(*pointing the opposite bed*) in labour for not positioning her legs well since I was laboring in the same room as that mother	The place is very clean and the food is very good	
**R54**	I was received well	I was not happy because my baby has not breastfed since yesterday and I have been telling the nurses and they are ignoring.I stayed for long during examination.The nurse who was examining me was not sure and I had to be examined by two Nurses ,When I was in labour ward I was not able to push and they slapped and pulled me	No	It is because of the way I was treated

		down the delivery bed telling me to go away if I did not want to deliver .I struggled alone and went back to the delivery bed.		
**R55**	There are very good services, the staff are good ,I did not take long before being attended to.	None	Yes<br>Because they offer very good services ,the hospital is not expensive	.
**R56**	None	None		No,I wouldn't recommend somebody to deliver here .The treatment I got was not good but it was may be due to my uncooperativeness
**R57**	I was happy with the way I was treated in this facility	None	Yes, I was very happy with the way I was treated during delivery so I would recommend a relative to come and deliver here	
**R58**	The staff really took good care of me from the time I arrived to the time of discharge	None	Yes, Because of good treatment	
**R59**	Nurses in this place are very cooperative, The Nurse rubbed my back when I was in lab our	None	Yes, The staff are very cooperative	
**R60**	None	The baby was almost coming out when I arrived at the facility gate and when my husband called out ,the male Nurse who was there ignored until the baby came out as we kept calling, RECC-During emergency Nurses should respond because I would have lost my baby had the driver not ran upstairs to ask for help.	Yes, Apart from neglecting me while I was delivering in the vehicle, the rest of the treatment was okay, The female Nurses are kind.	

R61		I did not have strength to push the baby and so many Nurses were around pressing my stomach to make the baby come out and I did not like it. Some Nurses come and tell you that if you joke we will throw you outside but others are good especially the male Nurses.	Yes, The goodness with this place is that I did not experience any beating as it happens in other hospitals	
**R62**	The services are good and the place is clean	I was being examined in the ward in the presence of other patients and I was not happy with that	Yes, Because the services are good	.
**R63**	The Nurses handled me well during delivery	They are mixing the mothers who have delivered with those who have not delivered so I felt bad when visitors come and the those who have delivered tell of their experience while me I was still going through lab our pains	Yes, The Nurses handle people well and no sharing of beds.	
**R64**	I was well supported by Nurses	There are a lot of vaginal examination .These frequent examinations result into a lot of pain	Yes, They support people well	
**R65**	Generally, the services are good; The Nurses are supportive and respond very fast. One cannot suffer and feels very safe	I was not happy because we were mixed with those who have already delivered yet me I was still going through lab our pains and I feel I was disturbing the ones who have delivered	Yes, The care here is good. The moment you enter, you are in safe hands.	
**R66**	The staff are treating people very well	None	Yes, Because the treatment here is good	
R67	I received good services	None	The nurses treated me well.	
R69	I liked the services I received from the facility.	None	I liked the services I received .	
R68	I liked how I was treated. The nurses helped me during delivery and 'took me slowly' when I was in pain.	None	The nurses were good to me	
R70	I liked the services I got.	None	I liked the treatment I got.	

R71	The treatment I got was good.	I did not like the fact that they have a male security guard who walks in and out of the ward that made me uncomfortable.	The facility is clean and the nurses fast in action.	
R72	I liked the way I was treated when I came to this health facility	The facility should provide each patient with her own bucket. I did not like the issue of sharing buckets for showering with other mothers	Yes<br>Because the services are good	
R73	I like the services I received		Yes<br>Because The Nurses treated me well	
R74	I like the way I was treated. In other hospitals I hear that Nurses beat people but here I was not beaten		Yes<br>Because I like how I was treated	
R75	I liked the services,the Nurses were good and took good care of me during delivery		Yes<br>The Nurses treated me well	
R76	I like how I was treated		Yes. Because of the good treatment	
R77	I was treated well		Yes<br>Because I was well treated	
R78	When you call Nurses, they respond fast and they keep on checking on us	Last time when I delivered here there were elderly Nurses who were a bit polite but these young Nurses shout at people when in labor<br>**RECC.** They should treat people politely	Yes, Because the services are good	

R79		I don’t like the fact that as soon as a mother comes,she is induced without being given a chance to labour natural.I also did not like harassment ,they don’t explain well and one is never given time to understandthe instructions.		

R80	I liked the way I was terated		Yes<br>Because of the treatment I received	
R81	The services were good		Yes<br>Because of good services	
R82	The treatment was good		Yes ,Because of the treatment I got	
R83	All services were good		Yes. Because I was treated well	
R 90				
R 91	The services I got were good		Yes The staff are good	
R92	The staff are good and caring and I felt they understood and paid attention to my problems		Yes, The Nurses are not rude as I hear in public facilities	
R 93	The staff were quick to serve me and kept on checking on me so I never felt neglected		Yes, The staff are quick to serve people and one will never feel neglected .	
R94	The services were good		Yes, because the services are good	
R95	Good facilities ,Good food and no sharing of beds		Yes, The staff respond quickly and offer good support	
R96	Good services to mothers and good food		Yes ,Because the hospital is clean and the services are good	
R97	The staff take good care of patients and talk nicely to people		Yes<br>The staff are good people and talk nicely to patients.	
R98	The staff coordinate well		Yes, Because the staff coordinate well.	
R99	The services were good		Yes<br>I like the services and good treatment	
R100	The doctors admitted me very fast ,they listen to us and I was encouraged emotionally		Yes, I was happy with the services I received	

